# Can predicting COVID-19 mortality in a European cohort using only demographic and comorbidity data surpass age-based prediction: An externally validated study

**DOI:** 10.1371/journal.pone.0249920

**Published:** 2021-04-15

**Authors:** Avishek Chatterjee, Guangyao Wu, Sergey Primakov, Cary Oberije, Henry Woodruff, Pieter Kubben, Ronald Henry, Marcel J. H. Aries, Martijn Beudel, Peter G. Noordzij, Tom Dormans, Niels C. Gritters van den Oever, Joop P. van den Bergh, Caroline E. Wyers, Suat Simsek, Renée Douma, Auke C. Reidinga, Martijn D. de Kruif, Julien Guiot, Anne-Noelle Frix, Renaud Louis, Michel Moutschen, Pierre Lovinfosse, Philippe Lambin

**Affiliations:** 1 The D-Lab, Department of Precision Medicine, GROW—School for Oncology, Maastricht University, Maastricht, The Netherlands; 2 Department of Neurosurgery, Maastricht University Medical Center, Maastricht, The Netherlands; 3 Department of Internal Medicine, Maastricht University Medical Center, Maastricht, The Netherlands; 4 Department of Neurology, Maastricht University Medical Center, Maastricht, The Netherlands; 5 Department of Neurology, Amsterdam University Medical Center, Amsterdam, The Netherlands; 6 Department of Anesthesiology and Intensive Care, St Antonius Hospital, Nieuwegein, The Netherlands; 7 Department of Intensive Care, Zuyderland Medical Center, Heerlen, The Netherlands; 8 Department of Intensive Care, Treant Zorggroep, Emmen, The Netherlands; 9 Department of Internal Medicine, VieCuri Medical Centre, Venlo, The Netherlands; 10 Department of Internal Medicine, Northwest Clinics, Alkmaar, The Netherlands; 11 Department of Internal Medicine, Flevoziekenhuis, Almere, The Netherlands; 12 Department of Intensive Care, Martiniziekenhuis, Groningen, The Netherlands; 13 Department of Pulmonary Medicine, Zuyderland Medical Center, Heerlen, The Netherlands; 14 Department of Respiratory Medicine, CHU of Liège, Liège, Belgium; 15 Department of Infectiology, CHU of Liège, Liège, Belgium; 16 Nuclear Medicine and Oncological Imaging, Department of Medical Physics, CHU of Liège, Liège, Belgium; Erasmus Medical Centre: Erasmus MC, NETHERLANDS

## Abstract

**Objective:**

To establish whether one can build a mortality prediction model for COVID-19 patients based solely on demographics and comorbidity data that outperforms age alone. Such a model could be a precursor to implementing smart lockdowns and vaccine distribution strategies.

**Methods:**

The training cohort comprised 2337 COVID-19 inpatients from nine hospitals in The Netherlands. The clinical outcome was death within 21 days of being discharged. The features were derived from electronic health records collected during admission. Three feature selection methods were used: LASSO, univariate using a novel metric, and pairwise (age being half of each pair). 478 patients from Belgium were used to test the model. All modeling attempts were compared against an age-only model.

**Results:**

In the training cohort, the mortality group’s median age was 77 years (interquartile range = 70–83), higher than the non-mortality group (median = 65, IQR = 55–75). The incidence of former/active smokers, male gender, hypertension, diabetes, dementia, cancer, chronic obstructive pulmonary disease, chronic cardiac disease, chronic neurological disease, and chronic kidney disease was higher in the mortality group. All stated differences were statistically significant after Bonferroni correction. LASSO selected eight features, novel univariate chose five, and pairwise chose none. No model was able to surpass an age-only model in the external validation set, where age had an AUC of 0.85 and a balanced accuracy of 0.77.

**Conclusion:**

When applied to an external validation set, we found that an age-only mortality model outperformed all modeling attempts (curated on www.covid19risk.ai) using three feature selection methods on 22 demographic and comorbid features.

## Introduction

Discovered in December 2019, the outbreak of COVID-19 is the most devastating pandemic in a century [1]. As of March 7, 2021, more than 116 million people have been infected globally, and about 2.6 million COVID-19 patients have died [2]. This pandemic has not only greatly threatened the lives and health of the world’s population but has also restricted global trade and economic development [3].

COVID-19 is a new disease caused by severe acute respiratory syndrome coronavirus 2 (SARS-CoV-2) which targets the angiotensin-converting enzyme 2 (ACE-2), with symptoms ranging from mild to critical [4]. The patients with a more severe illness are often more difficult to treat and have poor prognosis, including death [5]. While researchers have made unprecedentedly rapid progress in understanding the occurrence, progression, and treatment of the disease [6–8], it is still urgent to identify the risk factors for severe illness from COVID-19 and to protect the most vulnerable people. Previous studies have reported that older age, male gender, smoking and other conditions such as hypertension, diabetes, obesity, and chronic lung disease are the risk factors for severe illness or death [9–15]. However, to our knowledge, there are no publications that have attempted to predict disease severity or patient mortality using only such risk factors and then successfully validated such a model on an external cohort.

There is great value in having a predictive model for COVID-19 mortality that is based solely on demographics and underlying conditions, and which does not require blood test results or even initial symptoms. There are many beneficiaries from such a model. At the individual level, this can facilitate telemedicine for patients that have tested positive as well as inform citizens who have yet to contract COVID-19 of their potential risk, allowing them to take precautionary measures. At the government level, it can be used to implement smart lockdown approaches. As of March 2021, there are several vaccines that have obtained regulatory approval from various national agencies. However, given that the global population needs to be vaccinated against COVID-19, and the manufacturing limitations meaning that it is unlikely that the entire population can be inoculated in 2021, it is crucial that the available vaccines are distributed so as to have the maximum positive impact on public health. Such a predictive model would be a valuable addition to vaccine distribution decisions.

The aim of this study was to investigate potential risk factors and to develop prediction models for mortality based on demographics and underlying conditions and compare its performance to age alone. Ideally, a model would be desirable that can be applied to people who have not yet contracted COVID-19, so they can assess their risk level and take additional precautions compared to what is mandated by the government, e.g., not using public transportation, and doing all shopping online. This can also be applied by the authorities for rational prioritization of vaccination. We would also like a model that can be applied to people who have a positive test of COVID-19, and are undergoing a remote consultation with a physician to decide if they should visit the hospital. From a practical standpoint, however, such unbiased data is difficult to obtain, and thus our goal for this paper was to perform a proof-of-principle analysis using inpatient data from multiple European centers; the same analysis approach can then be applied to unbiased data, when available.

## Methods

### Study design and participants

The institutional ethics board of Amsterdam University Medical Center, The Netherlands, Maastricht University Medical Center, The Netherlands, and Liege University Hospital Centre, Belgium approved this retrospective study; the written informed consent was waived. All inpatients with confirmed COVID-19 from the nine Dutch hospitals (www.covidpredict.org) and consecutively enrolled from March 1, 2020 to July 1, 2020 comprised the training cohort. The contemporaneous data from Liege University Hospital was retrospectively collected for external validation. Patients with incomplete records (with more than half missing features), or under the age of 18 years, or lost to follow-up were excluded. This study can be classified as Type 3 (external validation) according to TRIPOD statement [16]. A confirmed case with COVID-19 was defined as a positive result of high-throughput sequencing or real-time reverse-transcriptase polymerase-chain-reaction assay for nasal and pharyngeal swab specimens. The end-point was death, and the follow-up period was 21 days after being discharged.

### Data collection

The data was derived from electronic health records collected during admission, including age, sex, smoking history, healthcare worker, alcohol use disorder, pregnancy, hypertension, diabetes, cancer, rheumatic diseases, autoimmune disorder, dementia, chronic obstructive pulmonary disease (COPD), asthma, chronic cardiac disease (CCD), chronic hematologic disease (CHD), chronic neurological disease (CND), chronic kidney disease (CKD), chronic liver disease (CLD), cachexia, Acquired Immune Deficiency Syndrome (AIDS), and organ transplant. The data was retrieved during July 12–16, 2020. Smoking history was categorized as current smokers, former smokers, and never smokers. Chronic cardiac disease included coronary artery disease, heart attack, and congenital heart disease. Chronic neurological disease contained Alzheimer’s disease, Parkinson’s disease, dystonia, Lou Gehrig’s disease, Huntington’s disease, neuromuscular disease, multiple sclerosis, and epilepsy. Chronic kidney disease was defined as glomerular filtration value < 60. Chronic liver disease comprised chronic hepatitis and liver cirrhosis.

### Statistical analysis

Missing data was imputed by using the missForest method based on the Random Forest (RF) algorithm [17]. Continuous data were summarized as median with interquartile range and categorical variables as frequency (%). Differences between the mortality and non-mortality groups were tested using the Mann-Whitney test for continuous data and χ^2^ test or Fisher’s exact test for categorical data. A two-sided p-value of less than 0.002 was regarded as statistically significant, chosen by applying a Bonferroni correction to the standard value of 0.05 to account for the 22 features being explored. This analysis was performed by using R for Windows (version 3.5.3). In addition, three feature selection approaches were used for predictive modeling, as described below.

#### LASSO-based feature selection

The least absolute shrinkage and selection operator (LASSO) with five-fold cross validation was used to select the optimal subset of predictive features. To check whether overtraining occurred in the LASSO approach, the training cohort was randomly spit into training and internal validation subsets (4:1). A logistic regression model and visual nomogram were built from the selected features. No imbalance adjustment was performed in the training cohort. Instead, the optimal classification threshold was chosen to maximize Youden’s J statistic. This threshold was also applied to the external validation set. The measures of model performance included the area under the receiver operating characteristic (ROC) curve (AUC), sensitivity, specificity, and balanced accuracy. This analysis was performed by using R for Windows (version 3.5.3). This model was compared against an age-only linear discriminant (LD) model in the external validation set.

#### Univariate feature selection using a novel metric

As described above, when deciding whether a feature differs between the mortality and non-mortality groups, the p-values of the relevant statistical tests were used. However, the fact that a feature differs between two groups does not necessarily mean that it can be used to discriminate between the two groups. To illustrate the limitation of a powerful binary feature (i.e., one with a large odds ratio) in terms of predictive modeling, let us consider two simple numerical examples. First, let us introduce some notation (exposed and non-exposed refer to the presence or absence of a risk factor) to define four categories of patients: dead and non-exposed (DN), healthy and non-exposed (HN), dead and exposed (DE), healthy and exposed (HE). The odds ratio is then given by: OR = (DE/HE)/(DN/HN)

In example 1, DE = 140, HE = 60, DN = 60, HN = 140. In this case, the exposed group (140+60) and non-exposed group (60+140) both have the same size. An example of such a risk factor could be male gender. The odds ratio is 5.44, and AUC is 0.7. If we were to build a linear discriminant classifier using just this feature, it would classify all the exposed patients as dead, and all the non-exposed patients as healthy. Thus, the sensitivity would be 140/(140+60) = 0.7, the specificity would be 140/(140+60) = 0.7, and the balanced accuracy would be 0.7.

In example 2, DE = 7, HE = 3, DN = 60, HN = 140. In this case, the exposed group (7+3) is 20 times smaller than the non-exposed group (60+140). An example of such a risk factor could be chronic liver disease. The odds ratio is still 5.44, but the AUC is only 0.54. If we were to build a linear discriminant classifier using just this feature, it would classify all the exposed patients as diseased, and all the non-exposed patients as healthy. Thus, the sensitivity would be 7/(7+60) = 0.10, the specificity would be 140/(140+3) = 0.98, and the balanced accuracy would be 0.54.

One way to decide whether a feature is good at distinguishing between two groups is to check its AUC. Of course, other metrics like sensitivity, specificity, positive predictive value, and the negative predictive value also matter, but if the AUC is no better than random, these other metrics will be poor as well. Checking that the AUC is above 0.5 is not sufficient to determine if a feature is useful, as higher values of AUC can happen randomly, and this becomes more likely for small training cohorts or when a large number of features are being considered.

The DeLong test can be used to determine if the AUC of a feature is better than another feature, a special case of which is a random feature. This fact was used to devise a novel metric for univariate feature selection. 1000 features were generated randomly as follows: for a training cohort of size *N*, each feature was a set of *N* numbers drawn randomly between 0 and 1 from a uniform distribution. The DeLong test was then performed (requiring p<0.05) to decide if the real feature under consideration was better than the randomly generated feature. We required that the real feature be better than a randomly generated feature at least 950 out of 1000 times, which we denoted as p_DeLong_ < 0.05. The features selected in this way were combined using a RF (200 trees, with the maximum number of decisions per tree limited to 3 to avoid overtraining). This model was compared against an age-only LD model in the external validation set. This feature selection method and the next method as well were implemented using MATLAB R2019a (MathWorks, Natick, MA).

#### Pairwise feature selection

As mentioned previously, 22 features were considered for inclusion, of which age was the only one known to be a powerful discriminator. In the pairwise approach, we built machine learning classifiers with just a pair of features at a time, where age was always part of each pair (e.g., age + sex, age + smoking history). Thus, there were 21 pairs considered (22 features–age). Two classifiers were used: LD (chosen for its simplicity) and RF (200 trees, with the maximum number of decision per tree limited to 5 to avoid overtraining). If either of these classifiers performed better than age alone, then the feature would be selected. To determine performance improvement, both AUC and balanced accuracy would need to be higher by at least 0.01 for the pairwise model compared to age alone. The reason for introducing this requirement was that when the training cohort is sufficiently large (over 1000 patients), even an improvement smaller than 0.01 in AUC could be statistically significant (i.e., DeLong test p-value < 0.05), and such a difference would have no impact in a real-world setting. All features selected in this way would then be combined using a RF. This model, if created despite the rigorous feature selection process (more restrictive than the first two feature selection methods), would then be compared against an age-only LD model in the external validation set.

## Results

### The characteristics of demography and complications

**Fig 1:**
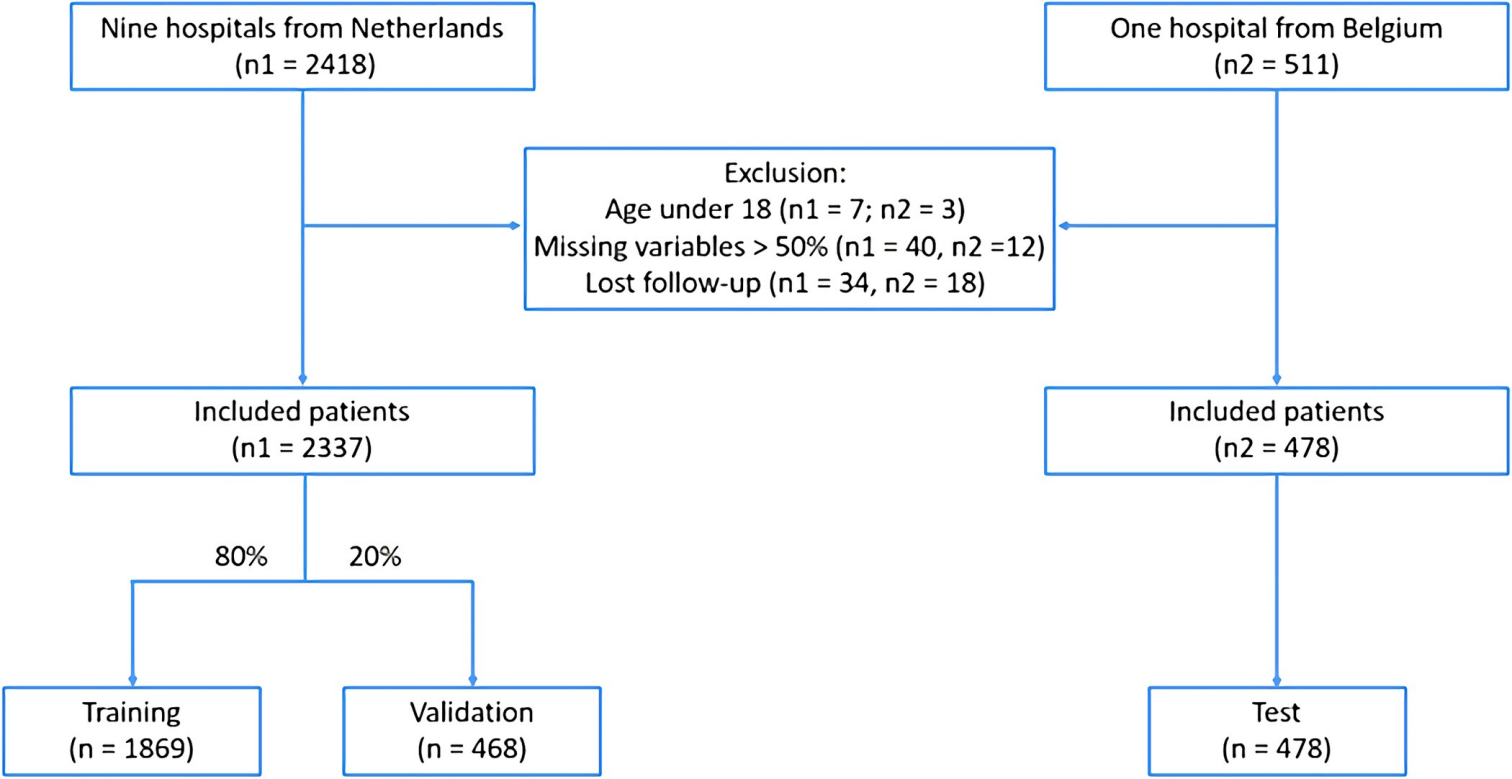
Flowchart of patient selection.

2929 COVID-19 patients were searched, of whom 114 patients were excluded from this study due to more than half missing variables (n = 52), being under the age of 18 (n = 10), and being lost to follow-up (n = 52) (Fig 1). Of the 2337 included COVID-19 patients from the Netherlands, 568 (24.3%) patients died. The characteristics of demography and complications are summarized in Table 1. Of the 478 included COVID-19 patients from Belgium, 41 (8.6%) patients died.

**Table 1:** Participant characteristics with missing values of COVID-19 patients (training cohort).

Characteristic	Missing Values, n (%)	Counts, n (%)
Died patients	0	568 (24.3)
Demography		
Age	0	Mean: 66.8 ± 14.4 y
Male	0	1471 (62.9)
Smoking	621 (26.6)	
Never smoking		861 (36.8)
Former smoker		738 (31.6)
Current smoker		117 (5.0)
Healthcare worker	104 (4.5)	98 (4.2)
Alcohol use disorder	453 (19.4)	50 (2.1)
Pregnancy	0	11 (0.5)
Complications		
Hypertension	7 (0.3)	1079 (46.2)
Diabetes	0	609 (26.1)
Rheumatic diseases	1 (0.04)	258 (11.0)
Autoimmune disorder	22 (0.9)	187 (8.0)
Dementia	13 (0.6)	99 (4.2)
Cancer	13 (0.6)	164 (7.0)
COPD	4 (0.2)	437 (18.7)
Asthma	7 (0.3)	226 (9.7)
CHD	3 (0.1)	87 (3.7)
CCD	3 (0.1)	706 (30.2)
CND	4 (0.2)	315 (13.5)
CLD	0	110 (4.7)
CKD	3 (0.1)	268 (11.5)
AIDS	4 (0.2)	10 (0.4)
Cachexia	0	33 (1.4)
Organ transplant	0	33 (1.4)

COPD, chronic obstructive pulmonary disease; CHD, chronic hematologic disease; CCD, chronic cardiac disease; CND, chronic neurological disease; CLD, chronic liver disease; CKD, chronic kidney disease; AIDS, Acquired Immune Deficiency Syndrome.

### Differences in groups

After imputation of missing values, the median age of mortality group was 77 years (interquartile range, 70–83), which was older than non-mortality group with a median age of 65 years (interquartile range, 55–75, p < 0.001). Smoking history showed significant difference between mortality and non-mortality group (p < 0.001). The proportion of healthcare workers in mortality group was lower than in non-mortality group (p < 0.001). The incidence of male gender (p < 0.001), hypertension (p < 0.001), diabetes (p < 0.001), dementia (p < 0.001), cancer (p = 0.001), COPD (p = 0.002), CCD (p < 0.001), CND (p < 0.001), and CKD (p < 0.001) in mortality group was higher than in non-mortality group. The detailed demographic and complication characteristics of the training cohort are shown in Table 2. A similar table for the external validation cohort can be found in Table A3 in S1 Appendix.

**Table 2:** Participant characteristics of mortality and non-mortality groups for COVID-19 patients after missing value imputation (training cohort).

	Non-mortality (n = 1769)	Mortality (n = 568)	p-value
Age (median [IQR])	65 [55, 75]	77 [70, 83]	<0.001
Sex, male (%)	1078 (60.9)	393 (69.2)	<0.001
Smoking (%)			<0.001
Never smoking	1004 (56.8)	250 (44.0)	
Former smoker	635 (35.9)	263 (46.3)	
Current smoker	130 (7.3)	55 (9.7)	
Healthcare worker (%)	112 (6.3)	6 (1.1)	<0.001
Alcohol use disorder (%)	61 (3.4)	27 (4.8)	0.164
Pregnancy (%)	11 (0.6)	0 (0.0)	0.076
Hypertension (%)	761 (43.0)	323 (56.9)	<0.001
Diabetes (%)	403 (22.8)	206 (36.3)	<0.001
Rheumatic diseases (%)	178 (10.1)	80 (14.1)	0.009
Autoimmune disorder (%)	147 (8.3)	43 (7.6)	0.659
Dementia (%)	47 (2.7)	61 (10.7)	<0.001
Cancer (%)	109 (6.2)	60 (10.6)	0.001
COPD (%)	306 (17.3)	132 (23.2)	0.002
Asthma (%)	183 (10.3)	44 (7.7)	0.073
CHD (%)	64 (3.6)	23 (4.0)	0.612
CCD (%)	461 (26.1)	247 (43.5)	<0.001
CND (%)	199 (11.2)	117 (20.6)	<0.001
CLD (%)	75 (4.2)	35(6.2)	0.068
CKD (%)	166 (9.4)	102 (18.0)	<0.001
AIDS (%)	10 (0.6)	0 (0.0)	0.131
Cachexia (%)	27 (1.5)	6 (1.1)	0.54
Organ transplant (%)	27 (1.5)	6 (1.1)	0.54

COPD, chronic obstructive pulmonary disease; CHD, chronic hematologic disease; CCD, chronic cardiac disease; CND, chronic neurological disease; CLD, chronic liver disease; CKD, chronic kidney disease; AIDS, Acquired Immune Deficiency Syndrome; IQR, interquartile range.

### LASSO-based feature selection

LASSO selected eight features including age, sex, smoking history, diabetes, dementia, cancer, CND, and CLD. Using these features, in the training set, the model had an AUC of 0.76 (95% CI: 0.74–0.78) with a sensitivity of 0.69, a specificity of 0.70, and a balanced accuracy of 0.70. In the internal validation dataset, the AUC was 0.76 (95% CI: 0.72–0.81), with a sensitivity of 0.75, a specificity of 0.68, and a balanced accuracy of 0.71. In the external validation set, the AUC was 0.84 (95% CI: 0.79–0.89), with a sensitivity of 0.61, a specificity of 0.83, and a balanced accuracy of 0.72. The nomogram is shown in Fig A1 in S1 Appendix. By comparison, an age-only LD model performed as follows in the external validation set: AUC = 0.85, sensitivity = 0.71, specificity = 0.84, balanced accuracy = 0.77; thus, the LASSO model did not perform better than the age-only model.

### Univariate feature selection

Table 3 shows the results of the univariate feature selection process. It includes AUC and p_DeLong_. For binary features, it also includes odds ratio and the p-value of the Fisher test. Although the Fisher test p-values were already mentioned in Table 2, they are included in Table 3 to show how different they are compared to our novel metric. Five features passed selection: age, smoking history, hypertension, diabetes, and CCD. The RF model based on these features performed as follows on the training cohort: AUC = 0.76, sensitivity = 0.73, specificity = 0.67, balanced accuracy = 0.70. On the external validation set, the results were: AUC = 0.85, sensitivity = 0.68, specificity = 0.84, balanced accuracy = 0.76. Thus, this model did not surpass the age-only model.

**Table 3:** Results of univariate feature selection in the training cohort using the novel metric p_DeLong_.

	AUC	p_DeLong_	OR	p-value
**Age**	**0.74**	**<0.001**	**-**	**-**
Sex	0.54	0.317	1.44	<0.001
**Smoking History**	**0.56**	**0.022**	**-**	**-**
Healthcare worker	0.53	0.555	0.16	<0.001
Alcohol use disorder	0.51	0.926	1.40	0.164
Pregnancy	0.50	0.947	0.00	0.076
**Hypertension**	**0.57**	**0.010**	**1.75**	**<0.001**
**Diabetes**	**0.57**	**0.012**	**1.93**	**<0.001**
Rheumatic diseases	0.52	0.805	1.47	0.009
Autoimmune disorder	0.50	0.970	0.90	0.659
Dementia	0.54	0.234	4.41	<0.001
Cancer	0.52	0.745	1.80	0.001
COPD	0.53	0.616	1.45	0.002
Asthma	0.51	0.893	0.73	0.073
CHD	0.50	0.949	1.12	0.612
**CCD**	**0.59**	**<0.001**	**2.18**	**<0.001**
CND	0.55	0.160	2.05	<0.001
CLD	0.51	0.904	1.48	0.068
CKD	0.54	0.227	2.11	<0.001
AIDS	0.50	0.946	0.00	0.131
Cachexia	0.50	0.958	0.69	0.54
Organ transplant	0.50	0.957	0.69	0.54

Selected features are in bold. COPD, chronic obstructive pulmonary disease; CHD, chronic hematologic disease; CCD, chronic cardiac disease; CND, chronic neurological disease; CLD, chronic liver disease; CKD, chronic kidney disease; AIDS, Acquired Immune Deficiency Syndrome; AUC, area under the Receiver Operating Characteristic curve; OR, odds ratio.

### Pairwise feature selection

Table 4 shows the results of pairwise feature selection. It shows AUC and balanced accuracy for each feature pair (i.e., age combined with one of the other 21 features), with two classifiers (LD and RF). No pair showed an improvement in AUC and balanced accuracy of at least 0.01, and therefore no feature passed selection. Thus, no predictive model could be built.

**Table 4:** Results of pair-wise feature selection in the training cohort.

	AUC (LD)	BA (LD)	AUC (RF)	BA (RF)
Age	0.742	0.685	0.753	0.694
Sex	0.748	0.684	0.757	0.693
Smoking History	0.745	0.684	0.754	0.688
Healthcare worker	0.743	0.685	0.754	0.692
Alcohol use disorder	0.743	0.686	0.754	0.690
Pregnancy	0.742	0.685	0.754	0.692
Hypertension	0.743	0.689	0.754	0.698
Diabetes	0.748	0.691	0.757	0.698
Rheumatic diseases	0.742	0.686	0.755	0.697
Autoimmune disorder	0.743	0.687	0.755	0.696
Dementia	0.747	0.690	0.755	0.690
Cancer	0.743	0.686	0.755	0.687
COPD	0.743	0.688	0.755	0.692
Asthma	0.743	0.686	0.755	0.690
CHD	0.742	0.685	0.753	0.693
CCD	0.741	0.688	0.752	0.690
CND	0.745	0.696	0.759	0.700
CLD	0.743	0.685	0.754	0.688
CKD	0.743	0.686	0.754	0.687
AIDS	0.743	0.686	0.753	0.696
Cachexia	0.744	0.687	0.754	0.694
Organ transplant	0.743	0.686	0.753	0.694

The first row shows the performance of age alone, and subsequent rows show the performance of a pair of features, where age is always one half of the pair, e.g., Sex means Age + Sex and Diabetes means Age + Diabetes. The third significant figure is included to show minute differences (not statistically significant). COPD, chronic obstructive pulmonary disease; CHD, chronic hematologic disease; CCD, chronic cardiac disease; CND, chronic neurological disease; CLD, chronic liver disease; CKD, chronic kidney disease; AIDS, Acquired Immune Deficiency Syndrome; AUC, area under the Receiver Operating Characteristic curve; BA, balanced accuracy; LD, linear discriminant; RF, random forest.

## Discussion

This multicenter study investigated the risk factors of mortality for the patients with COVID-19. Age, sex, smoking history, diabetes, dementia, cancer, chronic neurological disease, and chronic liver disease were selected by our LASSO feature selection.

Age is a recognized risk factor for the COVID-19 from current evidence [18]. Here, we found that the median age of mortality group was 77.0 years, which was significantly older than non-mortality group with a median age of 65.0 years. Our study found that males have a higher probability of dying when infected COVID-19, consistent with prior publications. Some researchers speculated this is due to higher smoking rates among males [19–21]. However, our predictive model includes both sex and smoking history, indicating that both of these play a contributing role and the dependence on sex cannot be explained away as an effect of smoking history.

Recent studies have reported hypertension, and diabetes were risk factors for the mortality of COVID-19 [22–24]. In this study, we found that the existence of hypertension and diabetes were more common on the mortality group. It is worth noting that hypertension and diabetes have a common association with increased ACE-2 receptor, and enhanced expression of ACE-2 receptor in adipose tissue has been found in recent publication [25]. However, it is unclear whether ACE-2-stimulating drugs or the complication itself increased risk of death for hypertension and diabetes. Patients with rheumatic diseases are at an increased risk of infection [26]. In this study, we found that the mortality group had more patients with rheumatic diseases than non-mortality group (14.1% vs 10.1%). However, this was not statistically significant after application of the severe Bonferroni correction for multiple hypothesis testing.

Epidemiological studies have shown a significant association between poor COVID-19 outcomes and dementia [27]. A study with 627 COVID-19 patients from Italy reported that the mortality rate was significantly higher in ones with dementia than the ones without [28]. Here, the dementia rate of mortality group was significantly higher than non-mortality group (10.7% vs. 2.7%). A previous study concluded that cancer treatments such as surgery, radiotherapy, and chemotherapy may weaken the immune system, which is detrimental for people if infected with COVID-19 [29]. Our findings were in agreement, as cancer rates in the mortality group were significantly higher than in the non-mortality group (10.6% vs 6.2%).

Furthermore, we found that several chronic diseases in lung, heart, kidney, and nervous system might increase the risk of mortality for COVID-19. The possible explanation is that these diseases have higher expression levels of ACE-2, which is associated with increased mortality of COVID-19 [30–32]. Current evidence indicated SARS-CoV-2 might have direct or indirect negative effect on the circulatory system such as abnormal clotting and vascular inflammation, though the detailed mechanism is still unclear [33]. Although the expression level of ACE-2 is low in central nervous system, some studies have put forward the neuro-invasive potentiality of SARS-CoV-2 that may potentially cause acute respiratory failure for patients with chronic neurological disease [34]. This may explain why chronic neurological disease is a risk factor for mortality caused by COVID-19.

Unlike previous published studies [35–37], our model used only the demographics and comorbidities of COVID-19 patients without any laboratory tests or medical images or even symptoms to predict the mortality. There was a recent publication [38] to predict in-hospital morality of COVID-19 patients using Italian data. Fine and Gray competing risks multivariate model (with discharge as a competing event) was used to develop a prediction rule for in-hospital mortality. However, their model included duration of symptoms before hospital admission and lactate dehydrogenase levels at hospital admissions, and thus cannot be applied for people who have yet to contract COVID-19. The aim of our approach was to find which features are likely to be most important when identifying patients with a high risk of mortality even before the patient has become symptomatic. However, in terms of model-building, we found that an age-only model could not be outperformed by any of our models in the external validation set. One can argue that if there is no overtraining, a model that performs better than an age-only model in just the training cohort may still be useful, since the performance in the external validation set is affected by factors such as differences in cohort characteristics and admission/treatment policy differences between The Netherlands and Belgium. The age-only model had an AUC of 0.74 in the training cohort (Table 3); compared to the LASSO model, which had an AUC of 0.76, it is slightly lower, but not enough to be clinically significant, and therefore not enough to justify the increase in model complexity (eight features vs one).

Of the 22 features we considered for model building, only two (age and smoking history) were not binary. Table 3 reveals that most binary features had modest odds ratios (around 2 or lower), and for the two exceptions (healthcare worker and dementia), the incidence of these factors within the cohort was low (about 5%). Thus, it is unsurprising that none of them had a high AUC (>0.6). When collecting data for a predictive model, whenever possible, it is better to use ordinal categorical variables (i.e., a scale) or continuous variables rather than convert them to binary variables. For example, when considering a pre-existing condition like alcohol use disorder, using a scale (say 0–4, tied to the average alcohol consumption per week) is superior to a simple binary feature (alcoholic vs not alcoholic). When considering a pre-existing condition like obesity, using a continuous variable like body mass index (BMI) is superior or a simple binary (BMI > = 30 vs BMI < 30). The reason is that the decision threshold of the predictive model can then be tuned based on the training data, whereas for a binary variable, no such tuning is possible.

The training cohort used in this paper has a large (>90%) overlap with another recent publication (Ottenhoff et al, [39]). However, that publication predicted mortality using premorbid and clinical presentation features in conjunction with laboratory and radiology values at admission. The aim was to achieve the highest possible predictive performance by leveraging the combination of this multifactorial data, which would then be used for effective patient triage during hospital admission. A secondary aim arose from the Dutch government’s firm opposition to using an age-based decision rule for ICU care because it is in violation of the constitution, which states that everyone should be treated equally. To contribute to this discussion, the effect of age on the best performing model was assessed, by retraining the model on the same feature set, while excluding age as feature. This paper, by sharp contrast, only uses demographic and comorbid data (since the ultimate aim is to make a predictive model for uninfected individuals), and tries to establish whether adding features to age benefits predictive performance. The team involved in designing this study and doing the statistical analysis has no overlap with the Ottenhoff et al publication. Furthermore, this study uses an external (non-Dutch) test cohort.

This study has certain limitations. The most important shortcoming is the biased nature of the dataset, since it only includes patients who were admitted to the hospital. However, we stress that obtaining an unbiased sample while still having the necessary follow-up data is a huge obstacle. For example, if using a smartphone app to obtain a cohort, it would be enormously challenging to know if a patient died after contracting COVID-19. We acknowledge that in the general population, comorbidities might be better prognostic factors than what was observed in our data. Second, the detailed breakdown of complications should be taken into account in the future studies, when larger datasets are available. This is particularly important because there is a wide spectrum of severity included under CCD and CND. Third, binary variables were included during data collection, which we argue should be avoided whenever possible. Fourth, our model was trained on Dutch patients and tested on Belgian patients. While the two cohorts were quite different (as seen by the drastically different mortality rates of 24.3% vs 8.6%), for a country that is sufficiently different from those included in this study, e.g., in terms of lifestyle or genetics, it is possible that our findings will not apply. Fifth, our study did not include BMI, which is now known to be an important risk factor [21, 22]. Unfortunately, due to the retrospective nature of the study, we were limited to only those features which were recorded by the various institutions in the absence of a well-defined protocol in the early months of the pandemic. We acknowledge that including BMI could potentially improve the performance of an age-only model. We have included BMI in the list of features for our ongoing prospective studies. Finally, RNA viruses can mutate rapidly and that could have an impact of the risk factor of the disease.

The reader may be surprised that even though Belgium has been reported in the news to have the highest per-capita mortality rate for COVID-19 in the world, the mortality rate in the Liege hospital is much lower than in the Dutch hospitals. The per-capita death rates in Belgium are based on counting deaths in hospitals and care homes, but including deaths in care homes that are suspected, not confirmed, as Covid-19 cases (i.e., no positive test required). Hence, there should not be any expectation of the per-capita mortality rates having any relationship to the rates observed in this study (which are based on hospital admissions and positive COVID-19 tests). We believe the driving factor for the difference in mortality rates between the Dutch and Belgian cohorts is age: the median age in the Dutch cohort was 68 years, whereas for the Belgian cohort, it was 58 years. Since there was no strictly-defined admission indication for the Dutch consortium, a comparison with the Belgian hospitalization criteria is not possible.

## Conclusions

We found some features of demographics and comorbidities to be significantly associated with death of COVID-19 patients. For people who have not been infected by COVID-19, a model based on such features could allow people with a potentially high risk of death to follow a much stricter set of preventive measures than what is mandated by the government, or even allow the government to enact a smart lockdown. For people who have been infected by COVID-19 but have yet to develop symptoms, such a model would help identify at-risk patients, who would then be encouraged by doctors to visit the hospital. The government could also use this model to design a vaccine distribution strategy that would maximize the positive impact on public health with the limited number of vaccines available at a given time. However, despite our multiple rigorous attempts to create such a model, we were unable to surpass the predictive performance of age alone in an external validation set. If the goal is to use a risk factor for predictive modeling, it is essential to mention the predictive performance of such a feature, including metrics such as AUC, sensitivity, specificity, and balanced accuracy. Researchers who are investigating potential risk factors for COVID-19 mortality should check to see if their findings actually improve predictive performance relative to age alone and not just state an odds ratio of the feature. While age is a strong prognosticator of mortality in this analysis, it is not possible to say whether this is due to senescence or due to the strong correlation of age with lifestyle-related comorbidities (e.g., hypertension and diabetes) in such a study. Future studies that take other biological age metrics into account besides chronological age are needed to address such questions.

## Supporting information

S1 Appendix Additional figures and tables that support the manuscript. (DOCX)

S1 Dataset Synthetic training cohort. (CSV)

S2 Dataset External validation cohort. (CSV)
