# Supplementary material for: Can predicting COVID-19 mortality in a European cohort using only demographic and comorbidity data surpass age-based prediction: An externally validated study
Source: PLoS One. 2021 Apr 15;16(4):e0249920. doi: 10.1371/journal.pone.0249920 (PMC8049248; doi:10.1371/journal.pone.0249920)

**Fig A1.** A nomogram of LASSO prediction model of mortality for COVID-19 patients.


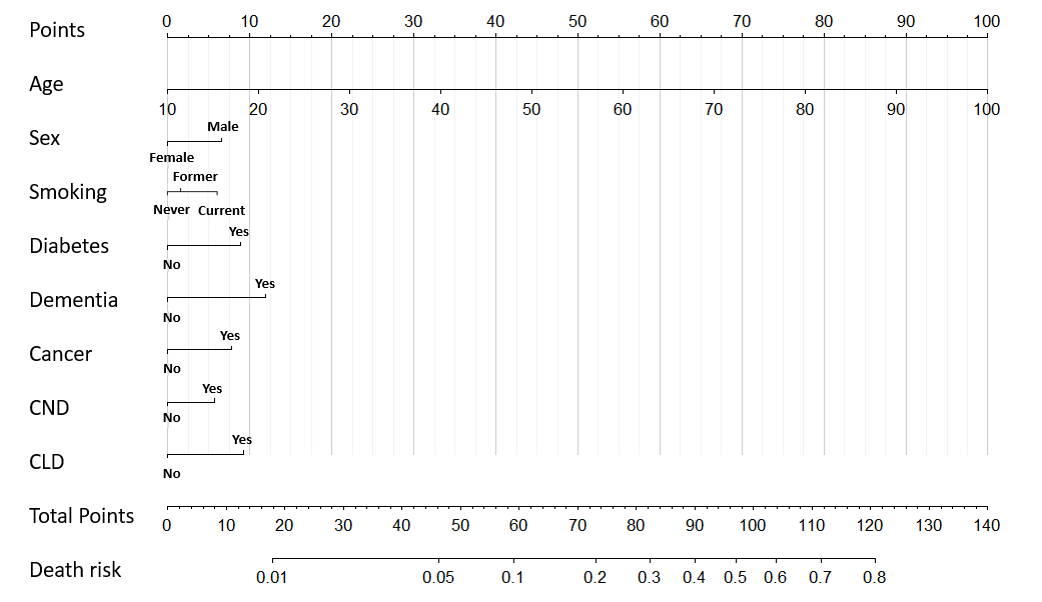


CND, chronic neurological disease; CLD, chronic liver disease.

**Synthetic data for training cohort**

As mentioned in the main manuscript, this was a retrospective study with training data from a Dutch consortium (www.covidpredict.org) and external validation data from a Belgian hospital (CHU Liege). The research ethics boards of the Dutch hospitals (specifically Amsterdam University Medical Center and Maastricht University Medical Center) and of CHU Liege approved the study and waived the informed consent requirement for conducting this analysis. However, in terms of data dissemination, while the CHU Liege ethics board approved this (after the data was irrevocably anonymized), the Dutch ethics boards did not agree to waiving the written informed consent requirement, even with anonymization. This is based on the Dutch principle that the patient owns their data, and informed consent cannot be waived when it comes to making such data public. Given the size of the Dutch dataset (2337 patients), obtaining written informed consent is unfeasible. As a result, we were able to share the anonymized data from CHU Liege, but could not share the Dutch training data. To address this issue, we created a synthetic population that mimics the Dutch cohort in terms of average values of patient features using the R function synthpop (doi:10.18637/jss.v074.i11). To evaluate how well the synthetic data matched the original data, we performed some comparisons of summary statistics. These are shown in Tables A1 and A2. Table A1 compares the counts for the 22 different features (for age, the comparison is of the median and IQR) between the original and synthetic data. It shows excellent overall agreement. Table A2 is the analog of Table 2, but with synthetic data. It shows the limitations of synthetic data: some features that had significant p-value in Table 2 are no longer significant in Table A2 (e.g., COPD, CKD).

**Table A1. Comparison of original (after imputation) and synthetic training cohort.**

| Characteristic | Synthetic Counts, n (%) | Original Counts, n (%) |
| --- | --- | --- |
| Died patients | 563 (24.1) | 568 (24.3) |
| Demography |  |  |
| Age | 68 [57, 78] y | 68 [58, 77] y |
| Male | 1519 (65.0) | 1471 (62.9) |
| Smoking |  |  |
| Never smoking | 1264 (54.1) | 1254 (53.7) |
| Former smoker | 904 (38.7) | 898 (38.4) |
| Current smoker | 169 (7.2) | 185 (7.9) |
| Healthcare worker | 103 (4.4) | 118 (5.0) |
| Alcohol abuse | 90 (3.9) | 88 (3.8) |
| Pregnancy | 9 (0.4) | 11 (0.5) |
| Complications |  |  |
| Hypertension | 1081 (46.3) | 1084 (46.4) |
| Diabetes | 593 (25.4) | 609 (26.1) |
| Rheumatic diseases | 251 (10.7) | 258 (11.0) |
| Autoimmune disorder | 188 (8.0) | 190 (8.1) |
| Dementia | 108 (4.6) | 108 (4.6) |
| Cancer | 164 (7.0) | 169 (7.2) |
| COPD | 437 (18.7) | 438 (18.7) |
| Asthma | 231 (9.9) | 227 (9.7) |
| CHD | 83 (3.6) | 87 (3.7) |
| CCD | 702 (30.0) | 708 (30.3) |
| CND | 325 (13.9) | 316 (13.5) |
| CLD | 118 (5.0) | 110 (4.7) |
| CKD | 260 (11.1) | 268 (11.5) |
| AIDS | 13 (0.6) | 10 (0.4) |
| Cachexia | 37 (1.6) | 33 (1.4) |
| Organ transplant | 32 (1.4) | 33 (1.4) |

For age, the median and IQR values are reported. COPD, chronic obstructive pulmonary disease; CHD, chronic hematologic disease; CCD, chronic cardiac disease; CND, chronic neurological disease; CLD, chronic liver disease; CKD, chronic kidney disease; AIDS, Acquired Immune Deficiency Syndrome.

**Table A2. Participant characteristics of mortality and non-mortality groups for COVID-19 patients in the synthetic training cohort (cf. Table 2).**

|  | Non-mortality (n = 1774) | Mortality (n = 563) | p-value |
| --- | --- | --- | --- |
| Age (median [IQR]) | 64 [55, 75] | 77 [69, 83] | <0.001 |
| Sex, male (%) | 1116 (62.9) | 403 (71.6) | <0.001 |
| Smoking (%) |  |  | 0.001 |
| Never smoking | 998 (56.3) | 266 (47.2) |  |
| Former smoker | 651 (36.7) | 253 (44.9) |  |
| Current smoker | 125 ( 7.0) | 44 ( 7.8) |  |
| Healthcare worker (%) | 93 ( 5.2) | 10 ( 1.8) | <0.001 |
| Alcohol abuse (%) | 69 ( 3.9) | 21 ( 3.7) | 1 |
| Pregnancy (%) | 6 ( 0.3) | 3 ( 0.5) | 0.457 |
| Hypertension (%) | 766 (43.2) | 315 (56.0) | <0.001 |
| Diabetes (%) | 413 (23.3) | 180 (32.0) | <0.001 |
| Rheumatic diseases (%) | 167 ( 9.4) | 84 (14.9) | <0.001 |
| Autoimmune disorder (%) | 139 ( 7.8) | 49 ( 8.7) | 0.534 |
| Dementia (%) | 57 ( 3.2) | 51 ( 9.1) | <0.001 |
| Cancer (%) | 112 ( 6.3) | 52 ( 9.2) | 0.023 |
| COPD (%) | 317 (17.9) | 120 (21.3) | 0.072 |
| Asthma (%) | 191 (10.8) | 40 ( 7.1) | 0.012 |
| CHD (%) | 62 ( 3.5) | 21 ( 3.7) | 0.794 |
| CCD (%) | 460 (25.9) | 242 (43.0) | <0.001 |
| CND (%) | 193 (10.9) | 132 (23.4) | <0.001 |
| CLD (%) | 91 ( 5.1) | 27 ( 4.8) | 0.826 |
| CKD (%) | 177 (10.0) | 83 (14.7) | 0.003 |
| AIDS (%) | 11 ( 0.6) | 2 ( 0.4) | 0.745 |
| Cachexia (%) | 33 ( 1.9) | 4 ( 0.7) | 0.078 |
| Organ transplant (%) | 28 ( 1.6) | 4 ( 0.7) | 0.147 |

COPD, chronic obstructive pulmonary disease; CHD, chronic hematologic disease; CCD, chronic cardiac disease; CND, chronic neurological disease; CLD, chronic liver disease; CKD, chronic kidney disease; AIDS, Acquired Immune Deficiency Syndrome; IQR, interquartile range.

**Table A3. Participant characteristics of mortality and non-mortality groups for COVID-19 patients in the external validation cohort (cf. Table 2).**

|  | Non-mortality (n = 437) | Mortality (n = 41) | p-value |
| --- | --- | --- | --- |
| Age (median [IQR]) | 55 [37, 68] | 79 [71, 85] | <0.001 |
| Sex, male (%) | 201 (46.0) | 25 (61.0) | 0.073 |
| Smoking (%) |  |  | 0.781 |
| Never smoking | 391 (89.5) | 36 (87.8) |  |
| Former smoker | 23 ( 5.3) | 3 ( 7.3) |  |
| Current smoker | 23 ( 5.3) | 2 ( 4.9) |  |
| Healthcare worker (%) | 126 (28.8) | 0 ( 0.0) | <0.001 |
| Hypertension (%) | 132 (30.2) | 27 (65.9) | <0.001 |
| Diabetes (%) | 70 (16.0) | 10 (24.4) | 0.188 |
| Dementia (%) | 2 ( 0.5) | 2 ( 4.9) | 0.039 |
| Cancer (%) | 16 ( 3.7) | 5 (12.2) | 0.026 |
| COPD (%) | 23 ( 5.3) | 5 (12.2) | 0.081 |
| Asthma (%) | 17 ( 3.9) | 0 ( 0.0) | 0.383 |
| CHD (%) | 2 ( 0.5) | 0 ( 0.0) | 1 |
| CCD (%) | 45 (10.3) | 16 (39.0) | <0.001 |
| CND (%) | 7 ( 1.6) | 1 ( 2.4) | 0.515 |
| CLD (%) | 10 ( 2.3) | 1 ( 2.4) | 1 |
| CKD (%) | 25 ( 5.7) | 11 (26.8) | <0.001 |

Some features that were available in the training cohort were not available in the external validation cohort, and have been omitted from this table. COPD, chronic obstructive pulmonary disease; CHD, chronic hematologic disease; CCD, chronic cardiac disease; CND, chronic neurological disease; CLD, chronic liver disease; CKD, chronic kidney disease; IQR, interquartile range.

**Fig A2.** Calibration curves of prediction models of mortality for COVID-19 patients: top row corresponds to the training set; bottom row corresponds to the external validation set. Left column corresponds to LASSO-based feature selection; right column to an age-only logistic regression model that has been included for comparison purposes only.


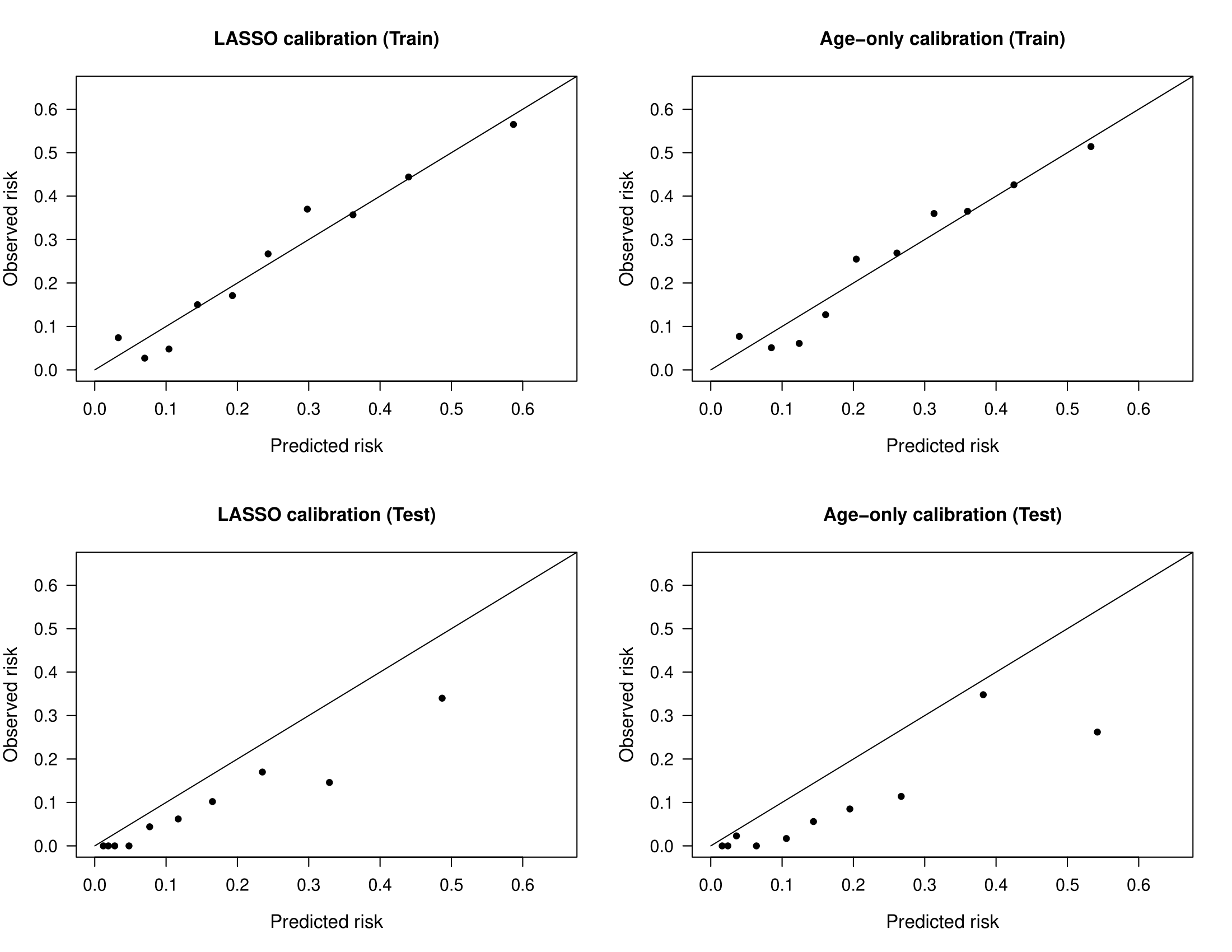

Supplement: S1 Appendix — (DOCX) [file pone.0249920.s001.docx]
